# Incessant ventricular tachycardia complicating heart failure in pregnancy

**DOI:** 10.4314/gmj.v59i1.5

**Published:** 2025-03

**Authors:** Dzifa Ahadzi, Hawa Malechi, Anita Avonsige, Issifu Amoaba, Shamrock Dokurugu Abdul-Latif, Abdul-Subulr Yakubu

**Affiliations:** 1 Department of Internal Medicine, Tamale Teaching Hospital, Tamale, Ghana; 2 Department of Obstetrics and Gynaecology, Tamale Teaching Hospital, Tamale, Ghana; 3 Department of Obstetrics and Gynaecology, School of Medical Sciences, University for Development Studies, Tamale, Ghana

**Keywords:** Ventricular tachycardia, Heart failure, Arrhythmia, Peripartum cardiomyopathy, Pregnancy

## Abstract

Cardiovascular diseases in pregnancy increase the risk of adverse maternal and foetal outcomes. Diagnosis and management of cardiovascular diseases, such as arrhythmias and heart failure in pregnancy, in resource-limited settings, is, however, challenging. We present a 31-year-old multiparous woman whose index presentation was for worsening symptoms of acute heart failure at 33 weeks of gestation. She had, however, had a prior history of breathlessness in a previous pregnancy for which she was diagnosed with peripartum cardiomyopathy. During this index clinical presentation, she was found to have dilated cardiac chambers with severe left ventricular systolic dysfunction and monomorphic ventricular tachycardia. Multidisciplinary team management resulted in successful cardioversion and satisfactory maternal and foetal outcomes.

**Funding:**

None declared

## Introduction

Arrhythmias in pregnancy are increasing in the developed world, though there is scarce similar data in the African region.^1^ Preconception, antepartum, intrapartum, and postpartum cardiovascular diseases (CVDs), including hypertension, pre-eclampsia, cardiomyopathies, valvular heart disease, and congenital heart diseases, can predispose to arrhythmias.^2^,^3^ Hemodynamic stresses associated with pregnancy, a history of arrhythmias, as well as undiagnosed CVDs, or poorly managed known CVDs can increase the arrhythmic risk in pregnancy. Heart failure (HF), one of the most common cardiovascular complications in pregnancy, can predispose to arrhythmias.^3^ In the developed world, atrial fibrillation appears to be the commonest tachyarrhythmia in pregnancy, whilst ventricular arrhythmias tend to be less common.^1^,^4^

Recognizing arrhythmias in pregnancy in resource-limited settings is challenging. The inability to recognise and accurately diagnose an abnormal rhythm, lack of basic equipment, and lack of skilled personnel can hamper a timely diagnosis of arrhythmias in pregnancy and its associated comorbidities and aetiologies like HF and structural heart disease, respectively.^5^ This case illustrates the systemic challenges that can occur in the management of arrhythmias in pregnancy in resource-limited settings.

It highlights the need for a multidisciplinary approach for optimal maternal and foetal outcomes.

## Case Report

A 31-year-old woman, gravida 5, para 4 (all alive), was referred to our facility from a primary care hospital at a gestational age of 33 weeks and 1 day. For this index clinical presentation, she had a 5-day history of worsening exertional dyspnoea, orthopnoea and paroxysmal nocturnal dyspnoea. Her past 4 pregnancies were carried to term and delivered by spontaneous vaginal delivery. She had experienced progressive dyspnoea following her fourth delivery, approximately 4 years prior, and reported a diagnosis of peripartum cardiomyopathy at that time. She had since not followed up for clinical care before the index pregnancy. There was no history of alcohol abuse or illicit drug use. There was no known family history of heart disease.

Initial assessment revealed an acutely breathless pregnant female who was pale and jaundiced with clinical evidence of fluid congestion. She was tachypneic and hypoxic (oxygen saturation of 83% on ambient air, which improved on supplemental oxygen). Her pulse was regular but fast (104 beats per minute (bpm)), and her blood pressure was 106/82mmHg with lateral displacement of the cardiac apex. She had an S3 gallop rhythm. Her abdomen was enlarged with a gravid uterus (symphysis fundal height of 32cm) and foetal heart rate of 133 bpm.

The patient was diagnosed with Acutely Decompensated Heart Failure in pregnancy secondary to Dilated Cardiomyopathy (DCM) and admitted to intensive care for emergent care and close monitoring.

**Figure 1:**
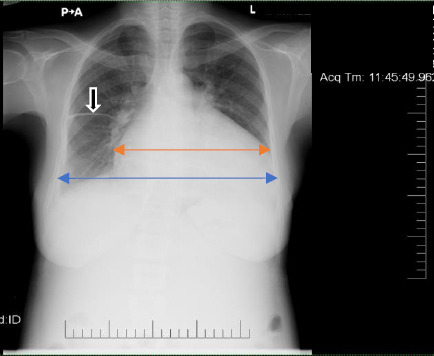
Chest Xray

**Figure 2:**
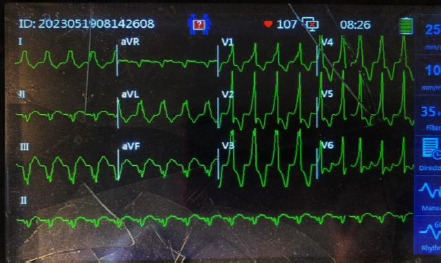
ECG rhythm on presentation Broad complex tachycardia at a rate of 107 bpm. Broad monophasic R wave in lead V1 with positive concordance in precordial leads and left axis deviation

A chest X-ray performed with an abdominal shield showed cardiomegaly with a curvilinear opacification in the horizontal fissure indicative of fluid accumulation (Figure 1). The cardiac monitor showed a broad complex tachycardia at a rate of 107 bpm (Figure 2). There was positive concordance in the precordial leads with a monophasic R wave in lead V1.

Despite the presence of a right bundle branch block (RBBB) )-like morphology in lead V1, there was left axis deviation. A monophasic R wave was observed in V1 and V6, unlike the triphasic R wave (rSR') in V1, which had an RS complex in V6, which is characteristic of a typical RBBB. Occasional sinus beats with narrow negative QRS complexes (capture beats) were observed during the recording. A monomorphic ventricular tachycardia (VT) was diagnosed. A bedside cardiac ultrasound showed dilated cardiac chambers with severe global hypokinesia (estimated LV ejection fraction of 20%), a central jet of mitral and tricuspid regurgitation with a plethoric non-collapsing inferior vena cava.

Chest X-ray showed cardiomegaly [ratio of the transverse diameter of the heart (red double arrow headline) to that of the thorax (blue double arrow headline) is greater than 0.5] and fluid in the horizontal fissure (black arrow).

Preliminary blood workup showed metabolic acidosis and mild hyponatremia (Table 1). The electrolyte panel and serum creatinine were otherwise normal. The liver function test showed elevated bilirubin, transaminases, gamma-glutamyl transferase, and alkaline phosphatase, likely due to hepatic congestion. A baseline thyroid function test was normal.

**Table 1:** Results of laboratory investigations

Investigation	Result	Reference range
**Full Blood count**		
Hemoglobin, g/dl	12.5	11.5-16.5
Platelet count, 10^9^/L	464.0	150.0-450.0
Total white cell count, 10^9^/L	6.5	4.0-12.0
**Renal Function**		
Sodium, mmol/l	134.0	135.0-150.0
Potassium, mmol/l	4.2	3.5-5.5
Chloride, mmol/l	100	98.0-107.0
Urea, mmol/l	6.4	2.0-7.0
Creatinine,	76	71.0-133.0
eGFR (ml/min/1.73m^2^)	>89	>89.0
Bicarbonate, mmol/L	15	22-29
**Liver Function Test**		
Total Bilirubin, µmol/l	69	3.42-20.52
Direct Bilirubin, µmol/l	40	0.0-5.0
Aspartate transaminase, U/L	101	0.0-32.0
Alanine transaminase, U/L	42.0	0.0-32.0
Alkaline phosphatase, U/L	272.0	35.0-105.0
Gamma-glutamyl transferase, U/L	84.0	<38.0
Total protein, g/dl	77.0	63.0-82.0
Albumin, g/dl	42.0	35.0-50.0
**Thyroid Function Test**		
TSH-Thyrotropin, uIU/ml	3.956	0.38-5.33
S-FT3 Triiodothyronine,pmol/l	5.6	3.5-7.8
S-FT4 thyroxine, pmol/l	14.7	7.9-18.5

Intravenous furosemide 40mg 12hrly and intramuscular dexamethasone 6mg twice daily were initiated to relieve fluid congestion and mature the foetal lungs, respectively. The patient was hemodynamically stable with normal blood pressures and incessant ventricular tachycardia at about 110 bpm. The foetal heart rate remained stable. She was diagnosed with Acute Decompensated Heart Failure (HFrEF (Heart Failure with reduced ejection fraction)) secondary to DCM with cardiohepatic syndrome associated with incessant monomorphic slow VT. Our differential diagnoses included (i) Recurrent Peripartum Cardiomyopathy and (ii) Heart Failure secondary to Peripartum Cardiomyopathy with persistent LV systolic dysfunction.

**Figure 3:**
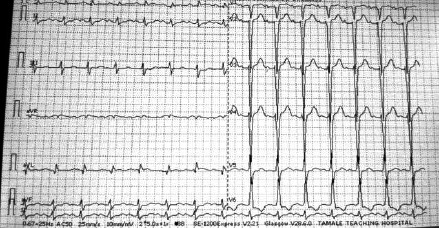
Post-cardioversion ECG Post-cardioversion ECG shows sinus rhythm with narrow QRS complexes, poor R wave progression and low voltage complexes in the precordial leads

Intravenous antiarrhythmic was chosen over DC cardioversion, though the patient was symptomatic of heart failure because she was haemodynamically stable, as evidenced by the absence of hypotension, shock, altered mental status or ischaemic chest pain. Intravenous amiodarone was given initially as a bolus of 150mg (150mg/10mls of 5% Dextrose water) over 10 minutes within reversion to sinus rhythm (Figure 3) within 10 minutes of bolus dosing. Continuous perfusion of amiodarone was then given at a rate of 1mg/minute over 6 hours. This was associated with improvement of her symptoms. The patient was counselled, and she consented to emergent caesarean delivery and bilateral tubal ligation under general anaesthesia.

A preterm baby with birth weight of 2.2 kilograms and Apgar scores of 6 and 8, was delivered and admitted to the neonatal intensive care unit. The baby's baseline TSH was normal.

The patient's postoperative course was complicated by postpartum haemorrhage that necessitated a blood transfusion. She otherwise remained hemodynamically stable, with no recurrence of VT, but had dyspnoea.

Cardiac ultrasound findings post-delivery were similar to baseline. The patient, together with her husband, was counselled on the effects of medical therapy on the baby in a shared decision-making approach. Considering the potential risks to the baby and benefits to the mother, the family opted to withhold breastfeeding. Post-delivery, oral lisinopril 2.5mg daily, spironolactone 12.5mg daily and amiodarone 600mg twice daily were initiated. A low dose of oral carvedilol 3.125mg twice daily was added when pulmonary congestion improved. The patient received prophylactic anticoagulation with subcutaneous enoxaparin sodium 40mg daily. This was switched to rivaroxaban 20mg daily at discharge on account of ease of administration after careful discussion in a shared decision-making process. Oral anticoagulation was considered on account of a high thrombotic risk, given the severity of LV systolic dysfunction. There was a significant improvement in her functional status (from NYHA Class IV to NYHA Class II). Mother and baby fared well, and she was discharged after 3 weeks on lisinopril 2.5mg daily, spironolactone 12.5mg daily, carvedilol 6.25mg twice daily, furosemide 40mg twice daily, amiodarone 400mg twice daily and rivaroxaban 20mg daily, and scheduled for follow-up.

## Discussion

Pregnancy is associated with significant physiologic changes, including cardiovascular, autonomic and hormonal changes.^6^ These changes, together with pre-existing CVD, pregnancy-related CVDs and previous history of an arrhythmia, can increase the arrhythmic risk during pregnancy.^1^

The approach to arrhythmias in pregnancy must take into consideration both mother and baby due to the high risk of adverse maternal and foetal outcomes.^1^ Limited evidence-based guidance on arrhythmia care in pregnancy due to a dearth of studies on the subject makes management even more challenging.^6^ However, a tailored case-by-case approach, with multidisciplinary management by cardiologists, obstetricians, neonatologists, and cardio-anesthesiologists, yields the best outcomes as demonstrated in this case.^6^

Whilst VT is rare amongst pregnant women in the United States, the true incidence of VT amongst pregnant women in countries in the African region is unclear.^1^ Ventricular tachycardia in pregnancy often occurs in patients with structural heart diseases such as cardiomyopathies and congenital heart disease.^7^ A good history, thorough physical examination and cardiac imaging are priceless in unmasking structural heart disease in pregnancy as illustrated in this case.

The patient had a previous diagnosis of PPCM but was lost to follow-up for 4 years, and her index presentation may be attributable to recurrence. Recovery of ventricular function post-PPCM is not uncommon, but there is a high recurrence rate in subsequent pregnancies with associated high long-term mortality.^8^ Factors responsible for the residual mortality risk in patients with recovered ventricular function are unclear. However, there is evidence to show that patients with PPCM with severe LV dysfunction have a very high risk of sudden cardiac death due to undiagnosed ventricular arrhythmias.^7^ In this case, recurrence manifested as acute heart failure associated with an incessant slow VT. Her arrhythmia was diagnosed through careful consideration of her ECG features as previously described. The timing of the onset of the arrhythmia is unclear, but diagnostic delays appeared in identifying the rhythm as VT before specialist consultation. This diagnostic delay may be due to a lack of confidence and skill in diagnosing life-threatening arrhythmias among healthcare givers, which may culminate in poor outcomes.^9^

The approach to our patient's management involved consideration of her hemodynamic status and local availability of IV antiarrhythmic medications. Of the IV therapeutic options for VT in pregnancy, IV amiodarone (a third-line agent) was the only intravenous antiarrhythmic medication available and was, therefore, our only option.^4^ Considering the potential adverse effects of amiodarone on both mother and baby, this option was discussed with the family in a shared decision-making approach before cardioversion. In a similar report of pregnancy-related ventricular tachycardia in a patient with PPCM in Saudi Arabia, IV amiodarone was used with good results.^10^

Optimal management of her HF was a key therapeutic goal. Unfortunately, financial constraints mitigated the use of Sacubitril/Valsartan and a sodium-glucose co-transporter 2 inhibitor (SGLT2i) for her HF post-delivery. Indications for anticoagulation in the context of dilated cardiomyopathy with HF include the presence of an intracardiac thrombus, venous thromboembolism, or atrial fibrillation.^11^ However, peripartum cardiomyopathy is uniquely associated with a high thrombotic risk due to the hypercoagulable state of pregnancy and the early postpartum period coupled with severe LV dysfunction.^12^ Anticoagulation may, therefore, be considered on a case-by-case basis, particularly in women with severely reduced LVEF of less than 35%.^12^ With counselling, the patient opted for oral anticoagulation on discharge and permanent contraception, given the possibility of recurrence in future pregnancies.

The management of VT and HF in pregnancy in low-resource settings can be challenging. Poor maternal health-seeking behaviour, late presentation, and limited numbers of trained specialists can result in diagnostic delays, as illustrated in this case. Limited availability of therapeutic options for arrhythmia care can lead to delays in the initiation of life-saving treatment with dire maternal and foetal outcomes. In this case, a continuous dialogue between the patient and the multidisciplinary team members was essential to achieving a positive outcome.

## Conclusion

Management of PPCM should include plans for long-term follow-up due to the risk of recurrence with subsequent pregnancies. Cardiomyopathies in pregnancy can be a predisposition for life-threatening ventricular arrhythmias, which can pose significant management challenges, particularly in low-resource settings. It is, however, possible to achieve good outcomes with a structured clinical approach, multidisciplinary care and shared decision-making.
